# Supplementary figures and images for: A master regulator of central carbon metabolism directly activates virulence gene expression in attaching and effacing pathogens
Source: PLoS Pathog. 2024 Oct 15;20(10):e1012451. doi: 10.1371/journal.ppat.1012451 (PMC11508082; doi:10.1371/journal.ppat.1012451)

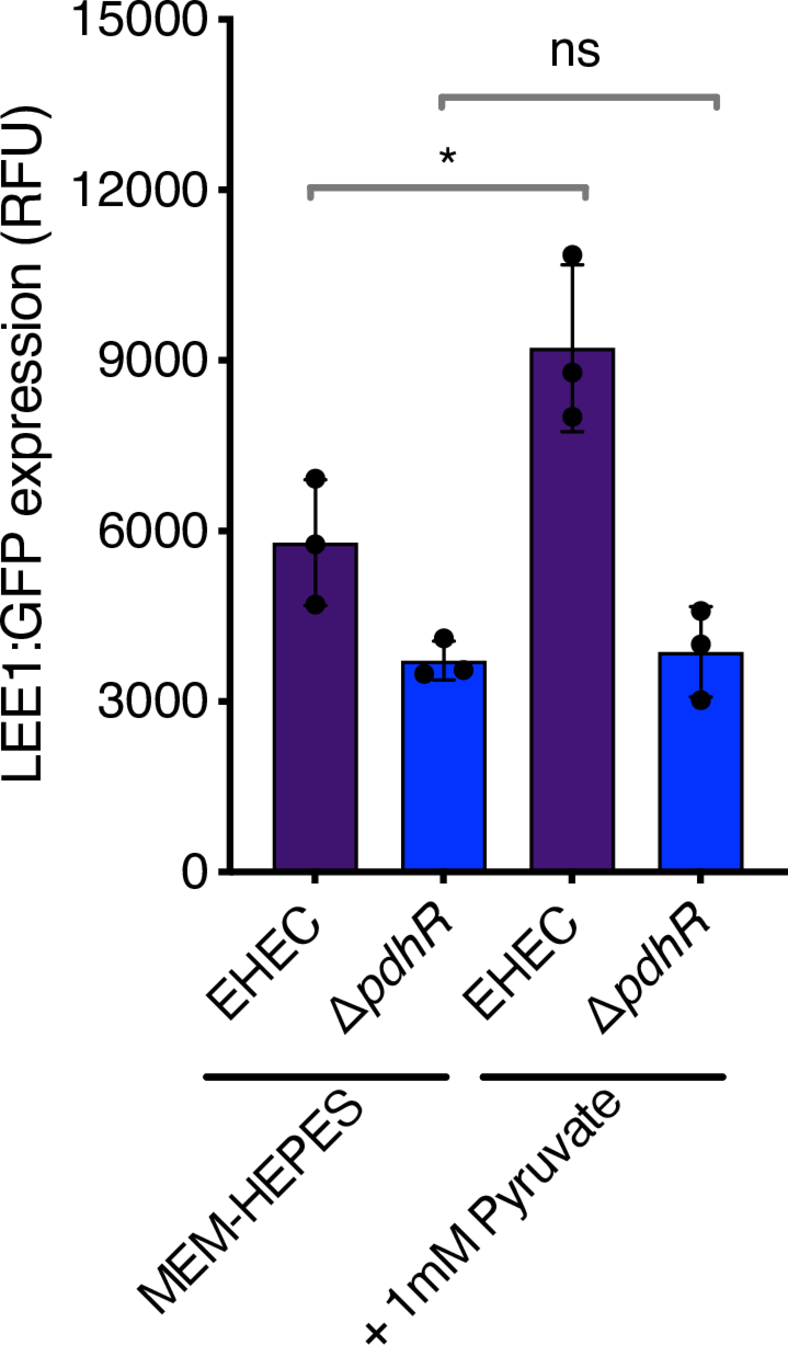

Supplement: S1 Fig — LEE-GFP reporter assay of EHEC and ΔpdhR cultured in MEM-HEPES alone or MEM-HEPES supplemented with 1 mM pyruvate. * and ns indicate P < 0.05 or not significant respectively, as determined by two-way ANOVA with Dunnett’s post-test. Error bars represent standard error of the mean. (TIF) [file ppat.1012451.s001.tif]

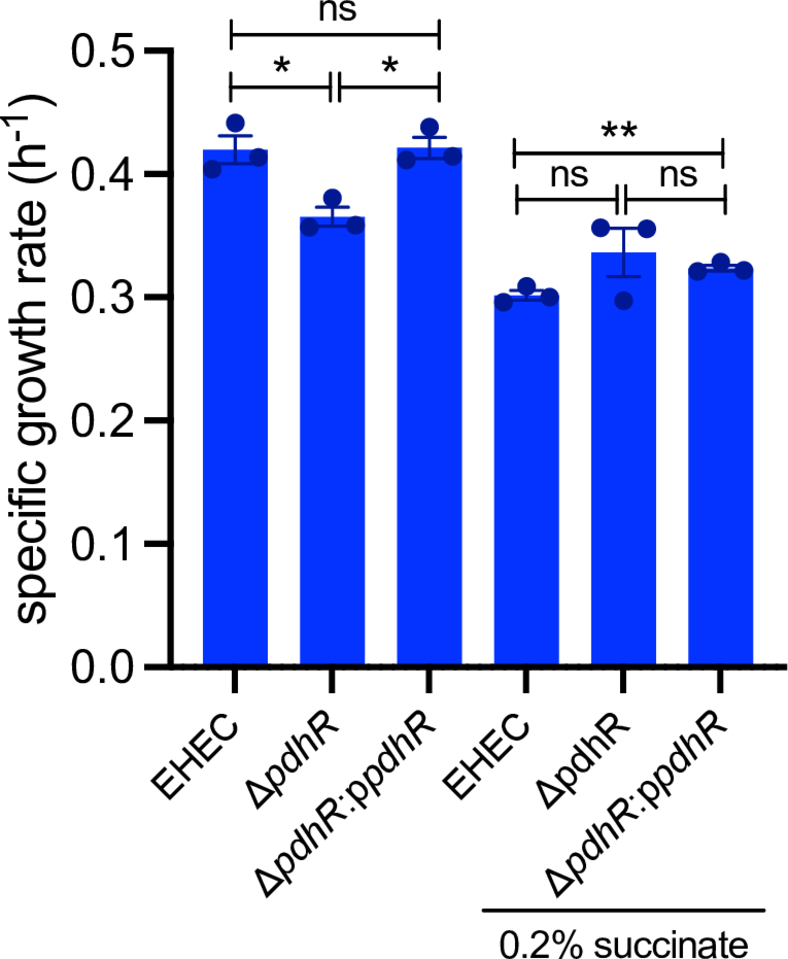

Supplement: S2 Fig — Specific growth rates were calculated between two and five hours using data from Fig 2A. Bars indicate means of three experiments with each experimental observation indicated by data points. Statistical significance was assessed by Ordinary one-way ANOVA with Tukey’s post-test for multiple comparisons (* and ns indicate P < 0.05 or not significant respectively). (TIFF) [file ppat.1012451.s002.tiff]

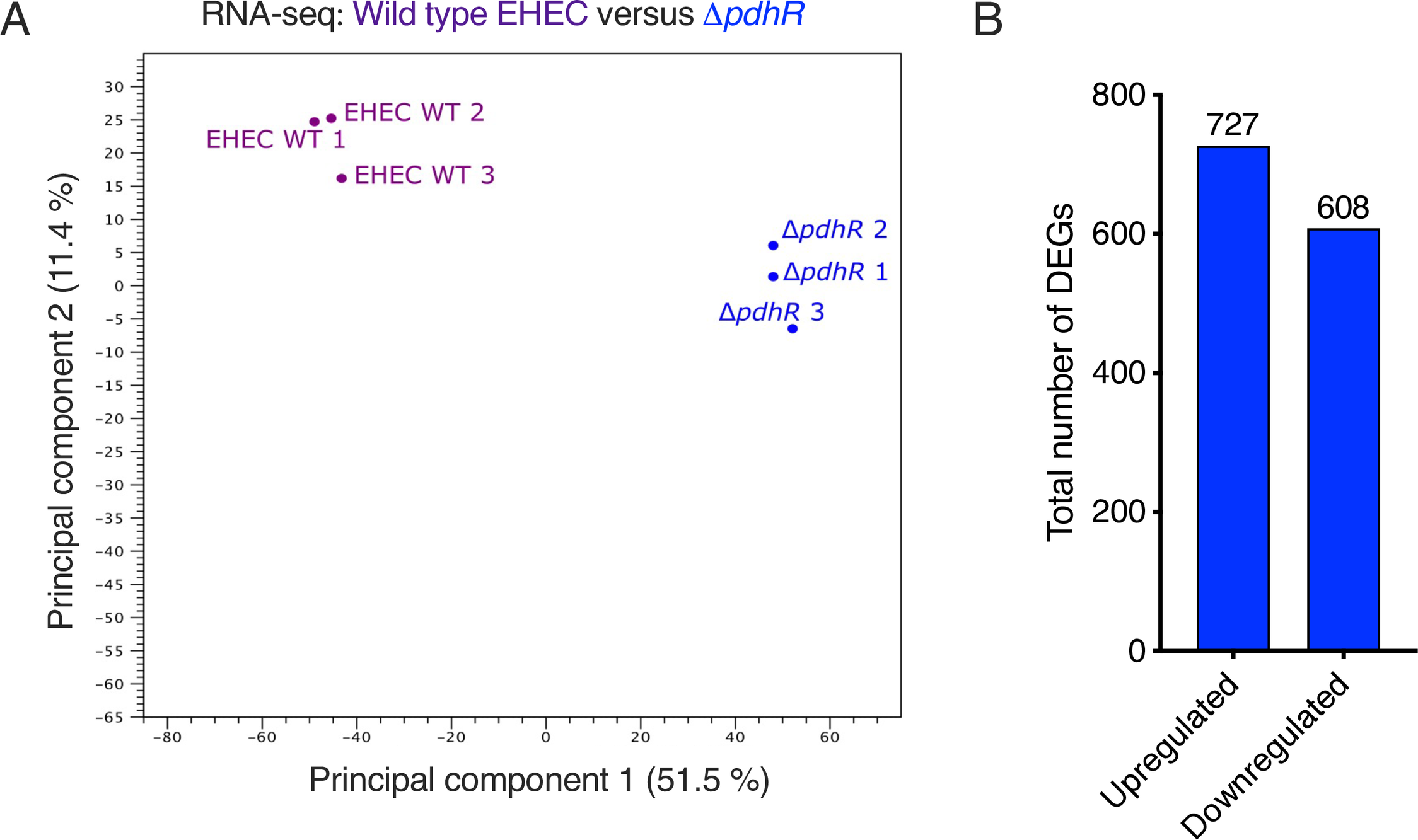

Supplement: S3 Fig — (A) Principal component analysis of the ΔpdhR versus EHEC transcriptomic data determined by RNA-seq., verifying the clustering of independently prepared biological replicates. (B) Total number of up and downregulated DEGs identified by RNA-seq. (TIFF) [file ppat.1012451.s003.tiff]

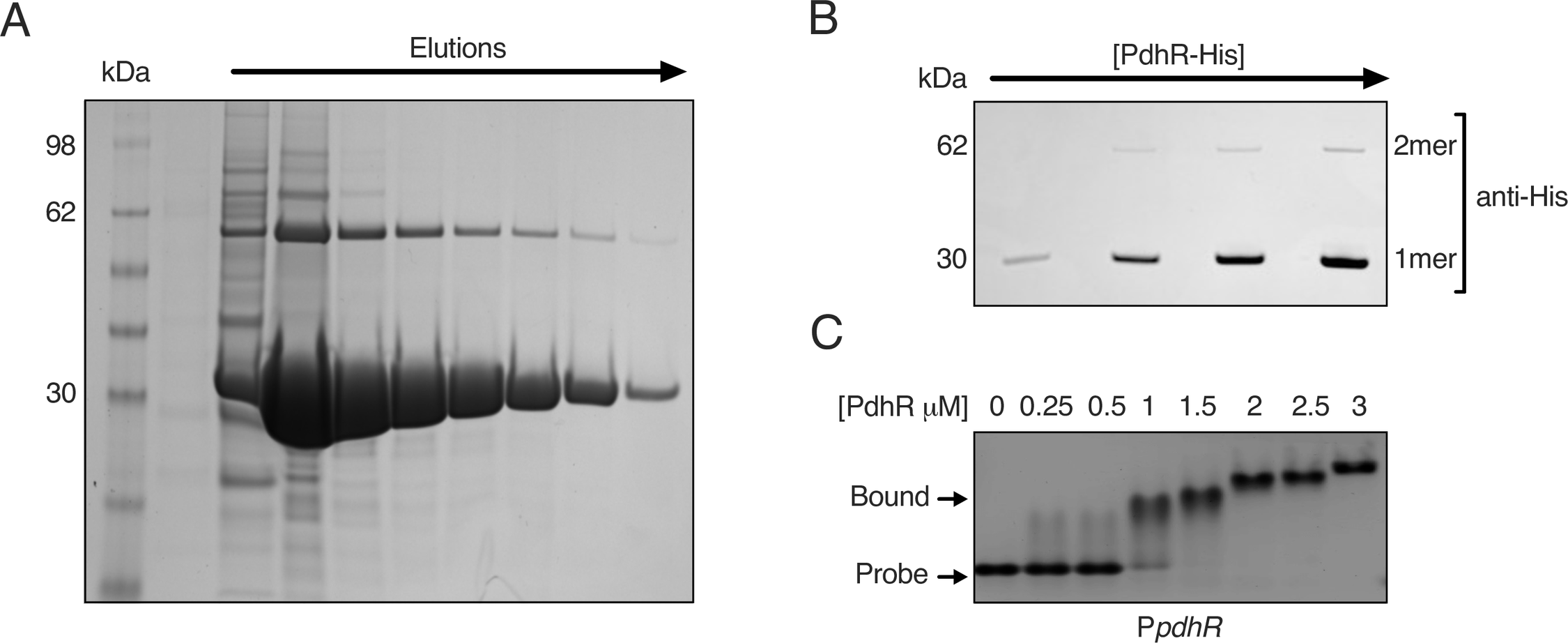

Supplement: S4 Fig — (A) SDS-PAGE analysis of over-expressed recombinant PdhR-his. The gel reveals elution of monomeric or dimeric PdhR in solution. (B) Immunoblot analysis of selected fractions from panel A confirming the bands as corresponding to monomeric or dimeric PdhR. (C) EMSA analysis of purified PdhR-his in complex with its own promoter region (PpdhR), verifying the functionality of the recombinant protein. (TIF) [file ppat.1012451.s004.tif]

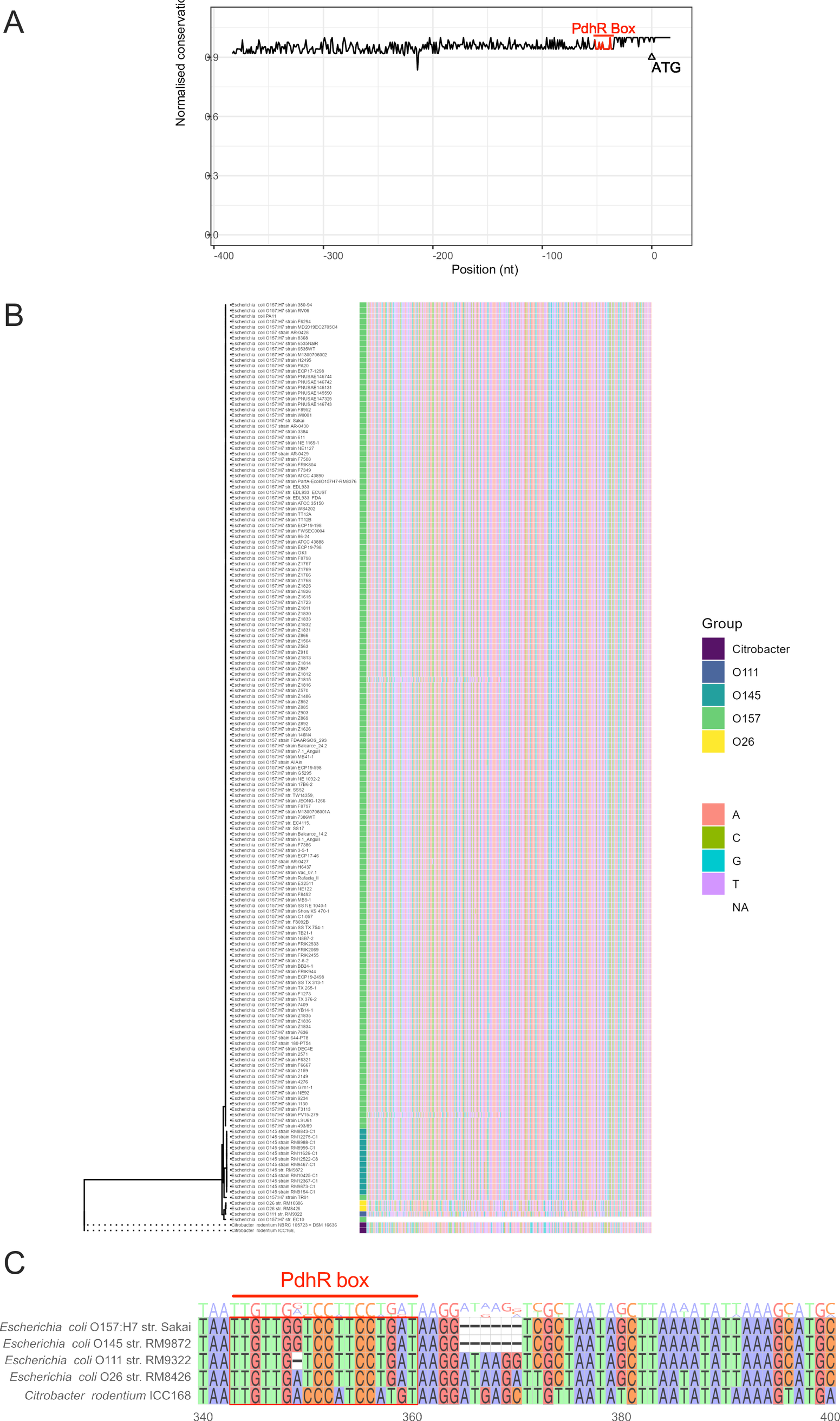

Supplement: S5 Fig — (A) Per-position conservation score of the unaligned upstream regions from all 168 assemblies in the dataset. (B) Core genome maximum-likelihood consensus tree of the 168 isolates queried here, assembled from an alignment of 707 core genes generated by Roary. This is lined-up with the unaligned LEE1 master regulatory region (upstream region of the ler start codon), illustrating how conserved the promoter region is amongst EHEC isolates. The non-LEE carrying EC-10 is included as a negative control. (C) ClustalW alignment of the region upstream of ler in C. rodentium and selected isolates of the E. coli O157, O145, O111, and O26 serotypes. The PdhR box is highlighted in red and the sequence logo of this motif is illustrated above the aligned sequence. (TIF) [file ppat.1012451.s005.tif]

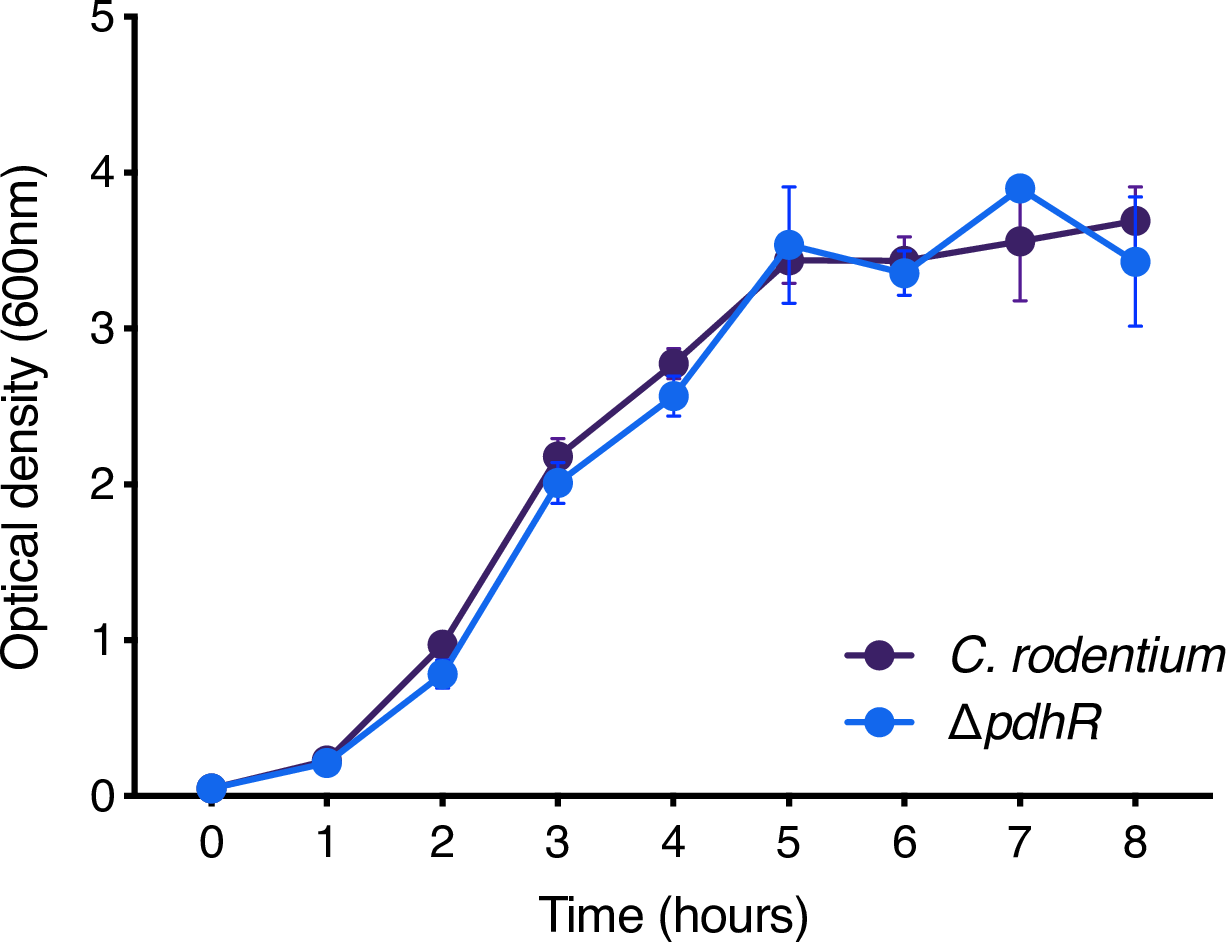

Supplement: S6 Fig — Growth curves depicting hourly optical density (600 nm) measurements of C. rodentium and the ΔpdhR mutant cultured in MEM-HEPES. Error bars represent standard error of the mean. (TIF) [file ppat.1012451.s006.tif]
