# Supplementary material for: A master regulator of central carbon metabolism directly activates virulence gene expression in attaching and effacing pathogens
Source: PLoS Pathog. 2024 Oct 15;20(10):e1012451. doi: 10.1371/journal.ppat.1012451 (PMC11508082; doi:10.1371/journal.ppat.1012451)
Supplement: S2 Table — (DOCX) [file ppat.1012451.s008.docx]

| **Strain** | **Description** | **Source** |
| --- | --- | --- |
| TUV93-0 | EHEC O157:H7 str. EDL933 Stx^-^ | Campellone *et al* [80] |
| ∆*yfeC* | TUV93-0 *yfeC* deletion mutant; Cm^R^ | This study |
| ∆*pdhR* | TUV93-0 *pdhR* deletion mutant; Cm^R^ | This study |
| ∆*bssS* | TUV93-0 *bssS* deletion mutant; Cm^R^ | This study |
| ∆*rcnR* | TUV93-0 *rcnR* deletion mutant; Cm^R^ | This study |
| ICC169 | *C. rodentium* O152 serotype; Nal^R^ | Petty *et al.* [81] |
| ICC169 ∆*pdhR* | ICC169 *pdhR* deletion mutant; Kan^R^ | This study |
| BL21(DE3) | Commercial overexpression strain | Invitrogen |
| DH5α | Commercial cloning and storage strain | Invitrogen |

**Table S2** – Bacterial strains used in this study
