# Supplementary material for: A master regulator of central carbon metabolism directly activates virulence gene expression in attaching and effacing pathogens
Source: PLoS Pathog. 2024 Oct 15;20(10):e1012451. doi: 10.1371/journal.ppat.1012451 (PMC11508082; doi:10.1371/journal.ppat.1012451)
Supplement: S3 Table — (DOCX) [file ppat.1012451.s009.docx]

| **Strain** | **Description** | **Source** |
| --- | --- | --- |
| pET-21a(+) | Plasmid for overexpression of  N-terminal 6xhis tagged proteins  (Kan^R^) | Roe lab inventory |
| pKD3 | Template plasmid for Lambda Red mutagenesis; Cm^R^ | Datsenko and Wanner [60] |
| pKD4 | Template plasmid for Lambda Red mutagenesis; Kan^R^ | Datsenko and Wanner [60] |
| pKD46 | Lambda Red recombinase expressing plasmid; temperature sensitive; Amp^R^ | Datsenko and Wanner [60] |
| p*rpsM*:GFP | pACYC184 based, *rpsM*:GFP transcriptional fusion | Roe *et al* [82] |
| LEE1:GFP | TUV93-0 LEE1 promoter region  cloned in-frame into pAJR70  (Cm^R^) | Roe *et al* [82] |
| LEE1P1:GFP | TUV93-0 LEE1 P1 distal promoter region  cloned in-frame into pAJR70  (Cm^R^) | This study |
| LEE1P2:GFP | TUV93-0 LEE1 P2 proximal promoter region cloned in-frame into pAJR70  (Cm^R^) | This study |
| p*pdhR* | pACYC184 derived *pdhR* complementation construct | This study |

**Table S3** – Plasmids used in this study
