# Supplementary material for: A master regulator of central carbon metabolism directly activates virulence gene expression in attaching and effacing pathogens
Source: PLoS Pathog. 2024 Oct 15;20(10):e1012451. doi: 10.1371/journal.ppat.1012451 (PMC11508082; doi:10.1371/journal.ppat.1012451)
Supplement: S4 Table — (DOCX) [file ppat.1012451.s010.docx]

**Table S4** – Primers used in this study

| **Primer** | **Description** | **Sequence** |
| --- | --- | --- |
| pdhR-Red-F | Forward for pdhR lambda red  mutagenesis in TUV93-0 | GAAATTGGTAAGACCAATTGACTTCGGCAAGTGGC  TTAAGACAGGAACTCGTGTAGGCTGGAGCTGCTTC |
| pdhR-Red-R | Reverse for pdhR lambda red  mutagenesis in TUV93-0 | TATGCGCTTGATTTACAACATCTTCTGGATAATTTT  TACCAGAAAAATCACATATGAATATCCTCCTTAG |
| pdhR-184-F | Check forward for pdhR lambda red mutagenesis in TUV93-0 | TGAAGTCAGCCCCATACGATTCAAGAATAATGGTA  TGCGGCA |
| pdhR-184-R | Check reverse for pdhR lambda red mutagenesis in TUV93-0 | CAATCCATGCCAACCCGTTCGGGAAACGTTCTGAC  ATGGG |
| bssS-Red-F | Forward for bssS lambda red mutagenesis in TUV93-0 | GCATTGAACCTCGAATAACGTTGTCTAGTAACACG  AATTAGGGGGCCATGGTGTAGGCTGGAGCTGCTTC |
| bssS-Red-R | Reverse for bssS lambda red mutagenesis in TUV93-0 | AATGGTAAAGGCACCGGTGAGGTGCCTTTTGGGT  GGATGGTCATGTCATGCATATGAATATCCTCCTTAG |
| bssS -184-F | Check forward for bssS lambda red mutagenesis in TUV93-0 | TGAAGTCAGCCCCATACGATTTATCGGTTATTGGC  GCGAC |
| bssS -184-R | Check reverse for bssS lambda red mutagenesis in TUV93-0 | CAATCCATGCCAACCCGTTCCGTCGGTCATAAGCA  CGTTT |
| yfeC-Red-F | Forward for yfeC lambda red mutagenesis in TUV93-0 | GTGCTATAAAATGAACTACTAATAGACCCACATACA  TTCAGGGAATTGTTGTGTAGGCTGGAGCTGCTTC |
| yfeC -Red-R | Reverse for yfeC lambda red mutagenesis in TUV93-0 | CATTCAGCCAGTTCTTCGGTGGTCATTTTATTGCG  TAATCTTTTCATACCCATATGAATATCCTCCTTAG |
| yfeC 184-F | Check forward for yfeC lambda red mutagenesis in TUV93-0 | TGAAGTCAGCCCCATACGATCCCGTCACGTAAAGC  TTGTC |
| yfeC -184-R | Check reverse for yfeC lambda red mutagenesis in TUV93-0 | CAATCCATGCCAACCCGTTCCAAACTTGCGTATCGA  CCAGA |
| rcnR-Red-F | Forward for rcnR lambda red mutagenesis in TUV93-0 | TAGATTAATAGTGCTATGATTTTTCATGTTCTTGTT  AACCAGGTGTTGCCGTGTAGGCTGGAGCTGCTTC |
| rcnR -Red-R | Reverse for rcnR lambda red mutagenesis in TUV93-0 | GTCACTTGTCCCTCTATTTATTGCCTCAACTACGGC  CATATTAGGCACTTCTAAGGAGGATATTCATATG |
| rcnR -184-F | Check forward for rcnR lambda red mutagenesis in TUV93-0 | TGAAGTCAGCCCCATACGATCGCCTGTTTAATGGT  GCCTT |
| rcnR -184-R | Check reverse for rcnR lambda red mutagenesis in TUV93-0 | CAATCCATGCCAACCCGTTCTGACGGTAGAAATCC  AGAGC |
| pdhR-C-Red-F | Forward for pdhR lambda red  mutagenesis in ICC169 | TAATTGGTAAGACCAATTGACTCCGGGCAAATGGC  TTAAGACAGGACATCGTGTAGGCTGGAGCTGCTTC |
| pdhR-C-Red-R | Reverse for pdhR lambda red  mutagenesis in ICC169 | TTTTGCGCTTTATTAACAACATCTTCTGGTAAACGT  ACTGCCAGAAAAAACATATGAATATCCTCCTTAG |
| pdhR-C-184-F | Check forward for pdhR lambda red mutagenesis in ICC169 | TGAAGTCAGCCCCATACGATCAGTGGAATGCACCT  GGTTT |
| pdhR-C-184-R | Check reverse for pdhR lambda red mutagenesis in ICC169 | CAATCCATGCCAACCCGTTCTGTCCCATTAAACTTT  CGTCGG |
| pAJR70_fwd | Forward for pACYC-184 linearisation | ACGATGCGTCCGGCGTAGAGGATCC |
| pAJR70_rev | Forward for pACYC-184 linearisation | GCCCTTGCTCACCATGGTACC |
| pdhR_fwd | Forward for pdhR for gibson assembly | ACGATGCGTCCGGCGTAGAGGATCCAACCCCTCTC  AATATGCAG |
| pdhR_rev | Reverse for pdhR for gibson assembly | GCCCTTGCTCACCATGGTACCCCAGTTGCTGCTCA  ATCAC |
| pAJR71_partial_fwd | Forward for pAJR71 linearisation | CGCTAATAGCTTAAAATATTAAAGC |
| pAJR71_partial_rev | Reverse for pAJR71 linearisation | CTCATGAGCGCTTGTTTC |
| LEE1_P1_fwd | Forward amplification of LEE1 promoter 1 from pAJR71 | CCGAAACAAGCGCTCATGAGCTGTGGCGCCGGTGA  TGC |
| LEE1_P1_rev | Reverse amplification of LEE1 promoter 1 from pAJR71 | AATATTTTAAGCTATTAGCGAAATCATCTCGTTAAC  AAACGACTTTAATAATTGCATTTCCATTTAG |
| LEE1_P2_fwd | Forward amplification of LEE1 promoter 2 from pAJR71 | CCGAAACAAGCGCTCATGAGTAATGTATTTTACACA  TTAGAAAAAAGAG |
| LEE1_P2_rev | Reverse amplification of LEE1 promoter 2 from pAJR71 | CTCATGAGCGCTTGTTTC |
| pdhR_NedI-F | Forward pdhR cloning into pET21 | GATATACATATGGCCTACAGCAAAATCCG |
| pdhR_XhoI-R | Reverse pdhR cloning into pET21 | GTGGTGCTCGAGATTCTTTCGTTGCTCCAGACG |
| ler qPCR F | Forward for *ler* gene expression using RT-qPCR | GAGCAGGAGATTCAAACTGT |
| ler qPCR R | Reverse for *ler* gene expression using RT-qPCR | TACCCCAGTTCTTGTAAGGT |
| eae qPCR F | Forward for *eae* gene expression using RT-qPCR | TTATTCATGGTTTTTGCACC |
| eae qPCR R | Reverse for *eae* gene expression using RT-qPCR | AAAACAATCCTAAACCAGCA |
| espZ qPCR F | Forward for *espZ* gene expression using RT-qPCR | CTGCAATAAATGGAAATGGT |
| espZ qPCR R | Reverse for *espZ* gene expression using RT-qPCR | AAAACTCTGACATTGCGACT |
| gapA_qPCR_F | Forward for housekeeping control (*gapA*) used in RT-qPCR | TTTCCGTGCTGCTCAGAAAC |
| gapA_qPCR_R | Reverse for housekeeping control (*gapA*) used in RT-qPCR | GGCCGTGAGTGGAGTCATAT |
| EMSA_LEE1_FWD | Forward for EMSA for validation of PdhR binding at LEE1 promoter region | GTATGGACTTGTTGTATGTGAATT |
| EMSA_LEE1_REV | Reverse for EMSA for validation of PdhR binding at LEE1 promoter region | GTATGGACTTGTTGTATGTGAATT |
| EMSA_grlA_FWD | Forward for EMSA for validation of PdhR binding at *grlA* promoter region | GGTTCCGATAGAAAGTCCTGGA |
| EMSA_grlA_REV | Reverse for EMSA for validation of PdhR binding at *grlA* promoter region | TAACTCTCCTTTTTCCGC |
| EMSA_PdhR_FWD | Forward for EMSA for validation of PdhR binding at *pdhR* promoter region | TGTGCACAGTTTCATGATTTCA |
| EMSA_PdhR_REV | Reverse for EMSA for validation of PdhR binding at *pdhR* promoter region | GCTGCTCAATCACATCGGAG |
| EMSA_Amp_FWD | Forward for EMSA for validation of PdhR binding at *amp* promoter region | CGCGGAACCCCTATTTGTTT |
| EMSA_Amp_REV | Reverse for EMSA for validation of PdhR binding at *amp* promoter region | AAGGGAATAAGGGCGACACG |
