# Supplementary material for: A master regulator of central carbon metabolism directly activates virulence gene expression in attaching and effacing pathogens
Source: PLoS Pathog. 2024 Oct 15;20(10):e1012451. doi: 10.1371/journal.ppat.1012451 (PMC11508082; doi:10.1371/journal.ppat.1012451)
Supplement: S5 Table — (DOCX) [file ppat.1012451.s011.docx]

**Table S5 - NCBI Accessions numbers of LEE-encoding isolates**

| Accession | Organism |
| --- | --- |
| GCF_000008865.2_ASM886v2 | Escherichia coli O157:H7 str. Sakai |
| GCF_000021125.1_ASM2112v1 | Escherichia coli O157:H7 str. EC4115, |
| GCF_000022225.1_ASM2222v1 | Escherichia coli O157:H7 str. TW14359, |
| GCF_000027085.1_ASM2708v1 | Citrobacter rodentium ICC168, |
| GCF_000730345.1_ASM73034v1 | Escherichia coli O157:H7 str. SS17 |
| GCF_000732965.1_ASM73296v1 | Escherichia coli O157:H7 str. EDL933 |
| GCF_000803705.1_ASM80370v1 | Escherichia coli O157:H7 str. SS52 |
| GCF_000978815.2_ASM97881v2 | Escherichia coli O157:H7 strain 7.1_Anguil |
| GCF_000978845.2_ASM97884v2 | Escherichia coli O157:H7 strain Rafaela_II |
| GCF_001307215.1_ASM130721v1 | Escherichia coli O157:H7 strain WS4202 |
| GCF_001558995.2_ASM155899v2 | Escherichia coli O157:H7 strain JEONG-1266 |
| GCF_001650275.1_ASM165027v1 | Escherichia coli O157 strain 180-PT54 |
| GCF_001650295.1_ASM165029v1 | Escherichia coli O157 strain 644-PT8 |
| GCF_001651925.2_ASM165192v2 | Escherichia coli O157:H7 strain FRIK2069 |
| GCF_001651945.2_ASM165194v2 | Escherichia coli O157:H7 strain FRIK2533 |
| GCF_001651965.2_ASM165196v2 | Escherichia coli O157:H7 strain FRIK2455 |
| GCF_001695515.1_ASM169551v1 | Escherichia coli O157:H7 strain FRIK944 |
| GCF_001753445.1_ASM175344v1 | Escherichia coli O157:H7 strain 1130 |
| GCF_001753465.1_ASM175346v1 | Escherichia coli O157:H7 strain 2149 |
| GCF_001753485.1_ASM175348v1 | Escherichia coli O157:H7 strain 8368 |
| GCF_001753505.1_ASM175350v1 | Escherichia coli O157:H7 strain 2159 |
| GCF_001753525.1_ASM175352v1 | Escherichia coli O157:H7 strain 9234 |
| GCF_001753545.1_ASM175354v1 | Escherichia coli O157:H7 strain 3384 |
| GCF_001753565.1_ASM175356v1 | Escherichia coli O157:H7 strain 4276 |
| GCF_001865295.1_ASM186529v1 | Escherichia coli O157:H7 strain PA20 |
| GCF_002208865.2_ASM220886v2 | Escherichia coli O157 strain FDAARGOS_293 |
| GCF_003112185.1_ASM311218v1 | Escherichia coli O26 str. RM8426 |
| GCF_003112225.1_ASM311222v1 | Escherichia coli O26 str. RM10386 |
| GCF_003112245.1_ASM311224v1 | Escherichia coli O111 str. RM9322 |
| GCF_003586065.1_EcRM9872 | Escherichia coli O145 str. RM9872 |
| GCF_003722195.1_ASM372219v1 | Escherichia coli O157:H7 strain TR01 |
| GCF_003966795.1_ASM396679v1 | Escherichia coli O157:H7 strain PV15-279 |
| GCF_004118915.1_ASM411891v1 | Escherichia coli O157:H7 strain C1-057 |
| GCF_005037735.1_ASM503773v2 | Escherichia coli O157:H7 strain FWSEC0004 |
| GCF_005885915.1_ASM588591v1 | Escherichia coli O157:H7 strain ECP17-1298 |
| GCF_005885955.1_ASM588595v1 | Escherichia coli O157:H7 strain ECP17-46 |
| GCF_007922655.1_ASM792265v1 | Escherichia coli O157:H7 strain ATCC 43888 |
| GCF_008462425.1_ASM846242v1 | Escherichia coli O157 strain Al Ain |
| GCF_008727135.1_ASM872713v1 | Escherichia coli O157 strain AR-0430 |
| GCF_008727155.1_ASM872715v1 | Escherichia coli O157 strain AR-0429 |
| GCF_008727175.1_ASM872717v1 | Escherichia coli O157 strain AR-0428 |
| GCF_008727195.1_ASM872719v1 | Escherichia coli O157 strain AR-0427 |
| GCF_009627495.1_ASM962749v1 | Escherichia coli O157:H7 strain TT12B |
| GCF_009650175.1_ASM965017v1 | Escherichia coli O157:H7 strain ATCC 43890 |
| GCF_009931235.1_ASM993123v1 | Escherichia coli O157:H7 strain FRIK804 |
| GCF_012915625.1_ASM1291562v2 | Escherichia coli O157:H7 strain MD2019EC2705C4 |
| GCF_013167135.1_ASM1316713v1 | Escherichia coli O157:H7 strain F8492 |
| GCF_013167155.1_ASM1316715v1 | Escherichia coli O157:H7 strain YB14-1 |
| GCF_013167175.1_ASM1316717v1 | Escherichia coli O157:H7 strain TX 376-2 |
| GCF_013167195.1_ASM1316719v1 | Escherichia coli O157:H7 strain TX 265-1 |
| GCF_013167235.1_ASM1316723v1 | Escherichia coli O157:H7 strain TB21-1 |
| GCF_013167275.1_ASM1316727v1 | Escherichia coli O157:H7 strain SS TX 754-1 |
| GCF_013167295.1_ASM1316729v1 | Escherichia coli O157:H7 strain SS TX 313-1 |
| GCF_013167315.1_ASM1316731v1 | Escherichia coli O157:H7 strain SS NE 1040-1 |
| GCF_013167335.1_ASM1316733v1 | Escherichia coli O157:H7 strain Show KS 470-1 |
| GCF_013167355.1_ASM1316735v1 | Escherichia coli O157:H7 strain OK1 |
| GCF_013167375.1_ASM1316737v1 | Escherichia coli O157:H7 strain NE92 |
| GCF_013167395.1_ASM1316739v1 | Escherichia coli O157:H7 strain NE122 |
| GCF_013167415.1_ASM1316741v1 | Escherichia coli O157:H7 strain NE1127 |
| GCF_013167435.1_ASM1316743v1 | Escherichia coli O157:H7 strain NE 1169-1 |
| GCF_013167455.1_ASM1316745v1 | Escherichia coli O157:H7 strain NE 1092-2 |
| GCF_013167475.1_ASM1316747v1 | Escherichia coli O157:H7 strain N8B7-2 |
| GCF_013167495.1_ASM1316749v1 | Escherichia coli O157:H7 strain LSU61 |
| GCF_013167515.1_ASM1316751v1 | Escherichia coli O157:H7 strain H6437 |
| GCF_013167535.1_ASM1316753v1 | Escherichia coli O157:H7 strain H2495 |
| GCF_013167555.1_ASM1316755v1 | Escherichia coli O157:H7 strain Gim1-1 |
| GCF_013167575.1_ASM1316757v1 | Escherichia coli O157:H7 strain G5295 |
| GCF_013167595.1_ASM1316759v1 | Escherichia coli O157:H7 strain F8952 |
| GCF_013167615.1_ASM1316761v1 | Escherichia coli O157:H7 strain F8798 |
| GCF_013167635.1_ASM1316763v1 | Escherichia coli O157:H7 strain F8797 |
| GCF_013167655.1_ASM1316765v1 | Escherichia coli O157:H7 str. F8092B |
| GCF_013167675.1_ASM1316767v1 | Escherichia coli O157:H7 strain F7508 |
| GCF_013167695.1_ASM1316769v1 | Escherichia coli O157:H7 strain F7386 |
| GCF_013167715.1_ASM1316771v1 | Escherichia coli O157:H7 strain F7349 |
| GCF_013167735.1_ASM1316773v1 | Escherichia coli O157:H7 strain F6667 |
| GCF_013167755.1_ASM1316775v1 | Escherichia coli O157:H7 strain F6321 |
| GCF_013167775.1_ASM1316777v1 | Escherichia coli O157:H7 strain F6294 |
| GCF_013167795.1_ASM1316779v1 | Escherichia coli O157:H7 strain F3113 |
| GCF_013167815.1_ASM1316781v1 | Escherichia coli O157:H7 strain F1273 |
| GCF_013167875.1_ASM1316787v1 | Escherichia coli O157:H7 strain E32511 |
| GCF_013168035.1_ASM1316803v1 | Escherichia coli O157:H7 strain DEC4E |
| GCF_013168055.1_ASM1316805v1 | Escherichia coli O157:H7 strain BB24-1 |
| GCF_013168075.1_ASM1316807v1 | Escherichia coli O157:H7 strain ATCC 35150 |
| GCF_013168095.1_ASM1316809v1 | Escherichia coli O157:H7 strain 86-24 |
| GCF_013168115.1_ASM1316811v1 | Escherichia coli O157:H7 strain 493/89 |
| GCF_013168135.1_ASM1316813v1 | Escherichia coli O157:H7 strain 17B6-2 |
| GCF_013168155.1_ASM1316815v1 | Escherichia coli O157:H7 strain 3-5-1 |
| GCF_013168175.1_ASM1316817v1 | Escherichia coli O157:H7 strain 2-6-2 |
| GCF_013168195.1_ASM1316819v1 | Escherichia coli O157:H7 strain 7636 |
| GCF_013168215.1_ASM1316821v1 | Escherichia coli O157:H7 strain 7409 |
| GCF_013168235.1_ASM1316823v1 | Escherichia coli O157:H7 strain 2571 |
| GCF_013168255.1_ASM1316825v1 | Escherichia coli O157:H7 strain 611 |
| GCF_013343595.1_ASM1334359v1 | Escherichia coli O157:H7 strain MB41-1 |
| GCF_013343635.1_ASM1334363v1 | Escherichia coli O157:H7 strain MB9-1 |
| GCF_014607535.1_ASM1460753v1 | Escherichia coli O145 strain RM10425-C1 |
| GCF_014607555.1_ASM1460755v1 | Escherichia coli O145 strain RM8995-C1 |
| GCF_014607575.1_ASM1460757v1 | Escherichia coli O145 strain RM12522-C8 |
| GCF_014622965.1_ASM1462296v1 | Escherichia coli O145 strain RM12275-C1 |
| GCF_014622985.1_ASM1462298v1 | Escherichia coli O145 strain RM8843-C1 |
| GCF_014623185.1_ASM1462318v1 | Escherichia coli O145 strain RM8988-C1 |
| GCF_014623205.1_ASM1462320v1 | Escherichia coli O145 strain RM11626-C1 |
| GCF_014623225.1_ASM1462322v1 | Escherichia coli O145 strain RM9154-C1 |
| GCF_014623245.1_ASM1462324v1 | Escherichia coli O145 strain RM9467-C1 |
| GCF_014623405.1_ASM1462340v1 | Escherichia coli O145 strain RM12367-C1 |
| GCF_014623425.1_ASM1462342v1 | Escherichia coli O145 strain RM9873-C1 |
| GCF_015353135.1_ASM1535313v1 | Escherichia coli O157:H7 strain Wll001 |
| GCF_016458885.1_ASM1645888v1 | Escherichia coli O157:H7 strain ECP19-198 |
| GCF_016458905.1_ASM1645890v1 | Escherichia coli O157:H7 strain ECP19-798 |
| GCF_016458925.1_ASM1645892v1 | Escherichia coli O157:H7 strain ECP19-598 |
| GCF_016458945.1_ASM1645894v1 | Escherichia coli O157:H7 strain ECP19-2498 |
| GCF_017164755.1_ASM1716475v1 | Escherichia coli O157:H7 strain Z1836 |
| GCF_017164775.1_ASM1716477v1 | Escherichia coli O157:H7 strain Z1835 |
| GCF_017164795.1_ASM1716479v1 | Escherichia coli O157:H7 strain Z1834 |
| GCF_017164815.1_ASM1716481v1 | Escherichia coli O157:H7 strain Z1833 |
| GCF_017164835.1_ASM1716483v1 | Escherichia coli O157:H7 strain Z1832 |
| GCF_017164855.1_ASM1716485v1 | Escherichia coli O157:H7 strain Z1831 |
| GCF_017164875.1_ASM1716487v1 | Escherichia coli O157:H7 strain Z1830 |
| GCF_017164895.1_ASM1716489v1 | Escherichia coli O157:H7 strain Z1826 |
| GCF_017164915.1_ASM1716491v1 | Escherichia coli O157:H7 strain Z1825 |
| GCF_017164935.1_ASM1716493v1 | Escherichia coli O157:H7 strain Z1816 |
| GCF_017164955.1_ASM1716495v1 | Escherichia coli O157:H7 strain Z1815 |
| GCF_017164975.1_ASM1716497v1 | Escherichia coli O157:H7 strain Z1814 |
| GCF_017164995.1_ASM1716499v1 | Escherichia coli O157:H7 strain Z1813 |
| GCF_017165015.1_ASM1716501v1 | Escherichia coli O157:H7 strain Z1812 |
| GCF_017165035.1_ASM1716503v1 | Escherichia coli O157:H7 strain Z1811 |
| GCF_017165055.1_ASM1716505v1 | Escherichia coli O157:H7 strain Z1769 |
| GCF_017165075.1_ASM1716507v1 | Escherichia coli O157:H7 strain Z1768 |
| GCF_017165095.1_ASM1716509v1 | Escherichia coli O157:H7 strain Z1767 |
| GCF_017165115.1_ASM1716511v1 | Escherichia coli O157:H7 strain Z1723 |
| GCF_017165135.1_ASM1716513v1 | Escherichia coli O157:H7 strain Z1766 |
| GCF_017165155.1_ASM1716515v1 | Escherichia coli O157:H7 strain Z1626 |
| GCF_017165215.1_ASM1716521v1 | Escherichia coli O157:H7 strain Z1615 |
| GCF_017165235.1_ASM1716523v1 | Escherichia coli O157:H7 strain Z1504 |
| GCF_017165255.1_ASM1716525v1 | Escherichia coli O157:H7 strain Z1486 |
| GCF_017165275.1_ASM1716527v1 | Escherichia coli O157:H7 strain Z910 |
| GCF_017165295.1_ASM1716529v1 | Escherichia coli O157:H7 strain Z903 |
| GCF_017165315.1_ASM1716531v1 | Escherichia coli O157:H7 strain Z887 |
| GCF_017165335.1_ASM1716533v1 | Escherichia coli O157:H7 strain Z892 |
| GCF_017165355.1_ASM1716535v1 | Escherichia coli O157:H7 strain Z885 |
| GCF_017165375.1_ASM1716537v1 | Escherichia coli O157:H7 strain Z866 |
| GCF_017165395.1_ASM1716539v1 | Escherichia coli O157:H7 strain Z869 |
| GCF_017165415.1_ASM1716541v1 | Escherichia coli O157:H7 strain Z852 |
| GCF_017165435.1_ASM1716543v1 | Escherichia coli O157:H7 strain Z570 |
| GCF_017165455.1_ASM1716545v1 | Escherichia coli O157:H7 strain Z563 |
| GCF_017357545.1_ASM1735754v1 | Escherichia coli O157:H7 strain TT12A |
| GCF_020423145.1_ASM2042314v1 | Escherichia coli O157:H7 strain Balcarce_24.2 |
| GCF_020423165.1_ASM2042316v1 | Escherichia coli O157:H7 strain 9.1_Anguil |
| GCF_020423185.1_ASM2042318v1 | Escherichia coli O157:H7 strain 146N4 |
| GCF_020423205.1_ASM2042320v1 | Escherichia coli O157:H7 strain Vac_07.1 |
| GCF_020423225.1_ASM2042322v1 | Escherichia coli O157:H7 strain Balcarce_14.2 |
| GCF_021166395.1_ASM2116639v1 | Escherichia coli O157:H7 strain 6535WT |
| GCF_021166415.1_ASM2116641v1 | Escherichia coli O157:H7 strain 6535NalR |
| GCF_021166435.1_ASM2116643v1 | Escherichia coli O157:H7 strain 7386WT |
| GCF_021184105.1_ASM2118410v1 | Escherichia coli O157:H7 strain M1300706002 |
| GCF_021184125.1_ASM2118412v1 | Escherichia coli O157:H7 strain M1300706001A |
| GCF_021278985.1_ASM2127898v1 | Citrobacter rodentium NBRC 105723 = DSM 16636 |
| GCF_022558925.1_ASM2255892v1 | Escherichia coli O157:H7 str. EC10 |
| GCF_022869945.1_ASM2286994v1 | Escherichia coli O157:H7 strain PartA-EcoliO157H7-RM8376 |
| GCF_025252345.1_ASM2525234v1 | Escherichia coli O157:H7 strain RV06 |
| GCF_025252445.1_ASM2525244v1 | Escherichia coli O157:H7 strain 380-94 |
| GCF_029590495.1_ASM2959049v1 | Escherichia coli O157:H7 str. EDL933 |
| GCF_029962285.1_ASM2996228v1 | Escherichia coli O157:H7 str. EDL933 |
| GCF_030908665.1_ASM3090866v1 | Escherichia coli O157:H7 strain PNUSAE145590 |
| GCF_030908685.1_ASM3090868v1 | Escherichia coli O157:H7 strain PNUSAE146742 |
| GCF_030908705.1_ASM3090870v1 | Escherichia coli O157:H7 strain PNUSAE146744 |
| GCF_030908725.1_ASM3090872v1 | Escherichia coli O157:H7 strain PNUSAE146131 |
| GCF_030908745.1_ASM3090874v1 | Escherichia coli O157:H7 strain PNUSAE146743 |
| GCF_031675355.1_ASM3167535v2 | Escherichia coli O157:H7 strain PNUSAE147325 |
| GCF_036923505.1_STEC28 | Escherichia coli PA11 |
