# Supplementary material for: A master regulator of central carbon metabolism directly activates virulence gene expression in attaching and effacing pathogens
Source: PLoS Pathog. 2024 Oct 15;20(10):e1012451. doi: 10.1371/journal.ppat.1012451 (PMC11508082; doi:10.1371/journal.ppat.1012451)
Supplement: Attachment — Submitted filename: Rebuttal_PdhR.docx [file ppat.1012451.s013.docx]

We would like to first thank the reviewers’ for taking the time to critique our manuscript and deliver their very constructive feedback. We appreciate the supportive comments and have done our best to address the concerns raised in the specific responses to each point below.

Reviewer's Responses to Questions

**Part I - Summary**
Please use this section to discuss strengths/weaknesses of study, novelty/significance, general execution and scholarship.

Reviewer #1: The manuscript describes how a central regulator of metabolism can be integrated into the virulence gene regulatory network of attaching and effacing pathogens. The regulator, PdhR, which controls gene expression in response to pyruvate availability within the intestine, directly regulates genes encoding a type III secretion system in enterohemorrhagic E. coli and the mouse pathogen Citrobacter rodentium.

Using a transcriptomics approach the authors find that PdhR controls expression of the global regulator Ler of the LEE PAI. They show in both EHEC and C. rodentium that LEE gene expression is diminished in a *pdhR* knockout strain, and that A/E lesions are reduced in a tissue culture model using *C. rodentium*. They show that purified PdhR protein binds to a conserved PdhR binding site and that colonization of the mouse intestine is less compared to wild type in the pdhR deletion strain.

In sum, the work gives a specific example as to how enteric pathogens can integrate central metabolism and host signaling events into virulence gene expression, which contributes to disease. The work is novel, important to the field, and draws clear conclusions based on multiple lines of evidence. A connection to central metabolism in the study of molecular pathogenesis is a critical part of our understanding, and this work pushes knowledge forward in this key area of inquiry.

Many thanks for the supportive feedback

Reviewer #2: This manuscript demonstrates that the TF PdhR is a regulator of the T3SS system in EHEC and *C. rodentium*. The authors previously reported that *pdhR* expression was increased in *C. rodentium* during mouse colonic infection. Here, the authors built on this finding to demonstrate a role for PdhR in regulating expression of ler, the master regulator of the T3SS-encoding LEE genes. Overall, this is a well-written manuscript that uses complementary approaches to justify conclusions. The findings are significant as the findings provide new insights into regulatory mechanisms important for EHEC and *C. rodentium* virulence. The findings also link metabolism and virulence. I have a couple of points that the authors should address:

Many thanks for the supportive feedback

1. A competition experiment for the EMSAs should be included. This is the gold-standard for demonstrating specificity of binding.

This was not included originally as the EMSAs were performed using post-stained gels, rather than radiolabelled DNA probes. Due to local legislation regarding use of radioisotopes we don’t have the capacity to repeat these experiments using radiolabelled DNA. It is therefore not possible for us to perform a typical competition control using an excess of unlabelled probe. As an alternative, we opted to test the functionality of the binding site by reporter assay after mutation of the PdhR box (Figure 4E) which clearly demonstrates the importance of the motif *in vivo*. To try and address the reviewers request, we have performed additional EMSA using the mutated version of the PdhR binding site as a probe. Mutation of the PdhR box reduces the ability of PdhR to bind to this site considerably. There remains some weak binding to the probe however this only occurs at very high protein concentrations. This could be due to the high protein concentration required, or a remaining interaction with the non-mutated part of the motif and compounded by the simplicity of an *in vitro* reaction, when other co-factors or interacting proteins are absent. We have included this result in the supplementary data (Lines 265-266; Figure S5).

2. How were the data normalized for the *in vivo C. rodentium* experiments showing ler expression? I did not see this described in the methods. The authors report decreased recovery of the pdhR strain compared to wt, and this would certainly lead to lower ler transcripts levels. The RT-qPCR are shown as relative values. What were the ct values for gapA? Differences in gapA transcript levels would affect relative ler expression using the analysis method described in the text.

Apologies for the lack of clarity. The data were normalised relatively as transcript levels for *gapA* were determined to not shift significantly between WT and *pdhR* mutant according to our RNA-seq data. We therefore used *gapA* levels of WT and *pdhR in vivo* to normalise our samples thus correcting for the differences in CFU abundance and compare *ler* expression levels between the strains.

3. Please include the C. rodentium pdhR growth curve in the supplemental data.

We apologise for the missing graph, which was inadvertently left out of our original submission. It is now included in the revised version as Figure S7.

Reviewer #3: This is a strong manuscript and very straightforward showing for the first time that the PdhR transcription factor that regulates pyruvate utilization in E. coli also regulates transcription of the LEE region and EHEC and C. rodentium, as well as virulence and pathogenesis during murine infection. This is important information to the field.

Many thanks for the supportive feedback

There are just some very minor weakness that are easily addressable with text changes or adding some extra information. I detailed them below.

**Part II – Major Issues: Key Experiments Required for Acceptance**
Please use this section to detail the key new experiments or modifications of existing experiments that should be absolutely required to validate study conclusions.

Generally, there should be no more than 3 such required experiments or major modifications for a "Major Revision" recommendation. If more than 3 experiments are necessary to validate the study conclusions, then you are encouraged to recommend "Reject".

Reviewer #1: None.

Reviewer #2:

1. A competition experiment for the EMSAs should be included. This is the gold-standard for demonstrating specificity of binding.

2. How were the data normalized for the in vivo C. rodentium experiments showing ler expression? I did not see this described in the methods. The authors report decreased recovery of the pdhR strain compared to wt, and this would certainly lead to lower ler transcripts levels. The RT-qPCR are shown as relative values. What were the ct values for gapA? Differences in gapA transcript levels would affect relative ler expression using the analysis method described in the text.

3. Please include the C. rodentium pdhR growth curve in the supplemental data.

Please see our responses to Reviewer 2 above.

Reviewer #3: No major issues or key experiments needed

**Part III – Minor Issues: Editorial and Data Presentation Modifications**
Please use this section for editorial suggestions as well as relatively minor modifications of existing data that would enhance clarity.

Reviewer #1:

1. Line 149. The authors use 1 mM pyruvate supplemented MEM-HEPES to illustrate PdhR-mediated signaling. Is the concentration of pyruvate in the mouse, or human intestine known, and how might this affect signaling within the intestine?

The reviewer is correct in alluding to the fact that endogenously produced pyruvate, bacterial secreted pyruvate and host-derived pyruvate could each influence LEE expression and that the concentration within the murine intestine will likely be a combination of these sources. Moreover, pyruvate concentration within the mouse will also likely be spatially variable making the question even more challenging to address. How this might exactly impact on LEE regulation *in vivo* is therefore difficult to answer for certain and certainly beyond the scope of the data we have presented in our manuscript. However, our experiment still manages to demonstrate the point that regardless of endogenous pyruvate being produced in the *pdhR* mutant, its deletion eliminates the signalling phenotype attributed to exogenous pyruvate. Thus, supporting the notion that the signalling occurs through this transcription factor.

2. Beginning on line 156, the authors discuss the previously observed growth defect caused by pdhR deletion in E. coli. While the colonization data presented in Figure 6 reach significance, how are the authors certain that the change in CFU’s per g of intestinal tissue don’t arise because of a growth defect of the deletion strain? While the authors also show diminished LEE gene expression, could a combination of events be occurring? Is a varied gene regulatory circuitry in C. rodentium compared to EHEC of concern with the observed results?

We tested the *in vitro* growth of the *pdhR* deletion in *C. rodentium* and saw no effect on fitness relative to the WT (Supplementary data Figure S7 now included in our revision, as requested also by reviewer 1). This contrasts with the *pdhR* deletion in EHEC, which has a significant growth defect. Of course, *in vivo* it is possible that the lack of *pdhR* in *Citrobacter* may result in the observed defect through a combination of LEE regulation and insufficient metabolic regulation. Our *in vivo* gene expression data (which is normalised for relative housekeeping expression) would suggest that, in the mutant, LEE expression is reduced. We hypothesise that this would be a major factor influencing total colonisation numbers but agree this may not be the sole reason. We have clarified this point in the revised manuscript (Lines 324-325 and line 436).

3. Line 207. It is unclear what the authors are referring to by the phrase “translation membrane composition,” “with other enriched terms corresponding to less specific processes such as translation membrane composition likely mirroring the core function of PdhR.” Please clarify.

Thank you for pointing this out. It should read “translation and membrane composition” referring to these two very general GO terms identified in our analysis. We have now corrected this in the text (Line 224).

Reviewer #2: n/a

Reviewer #3:

The emphasis on pyruvate regulation of LEE expression through PdhR should be toned down a bit. The only data here is Figure S1 and although significant it is not that striking. The data on PdhR regulation per se is very strong.

Thank you for the feedback, which we appreciate, but we would like to leave the discussion as it stands. Given our very recent study (PMID: 38796512) and the work of others (PMID: 27879335) linking pyruvate to LEE regulation, we felt it was important to highlight this as our current data presented in this paper is the first evidence of how pyruvate may be directly sensed by the transcription factor in question to regulate the T3SS and is therefore highly relevant to a number of ways in which pyruvate may be encountered or generated.

The authors should add more details in methods on the environmental conditions the cultures were grown. I got 37oC in MEM-HEPES. However, it is important to add the growth phase (early, mid, late log or stationary?) as well as oxygen tension (aerobic, anaerobic or microaerobic conditions). All of these are known to affect LEE expression, and it is important for the community to know when this regulation is occurring.

Thank you for highlighting this shortcoming. We have now modified the text in the methods to include all these details (Lines 450-452 and Line 500).

Growth curves on Figure 2A need p Values and the calculation of doubling rates

This data has now been included as Figure S2 in the revised manuscript.

Can the authors please clarify if the subsequent EHEC experiments were performed with or without succinate?

The subsequent EHEC experiments were performed with succinate as it allowed us to evaluate the effect of PdhR on LEE expression and function without the results being biased by a major growth defect under these conditions.

Fig. 2D, The first FAS panel is great, I don't see the need for the GFP or Rodamine panels, instead it would be batter to have an inlet or an extra panel of a higher magnification of the FAS images.

Thank you for the suggestion. We chose to present the data this way as we feel it makes it easier to identify the areas of condensed actin underneath where the bacteria are attached (made even clearer by displaying each channel in parallel).

Fig. 5A would be nice to add the complement to the transcription studies, since they obviously have that on the FAS experiments

Thank you for the suggestion. The results of the FAS are dictated by the levels of LEE transcription and so we felt that phenotypic complementation at the cellular level was sufficient in this context to make the point. In combination with data from figure 4E validating the regulatory mechanism and effect of PdhR at a specific binding site, this collectively supports our findings.

Fig. 5C on FAS same comment as per FAS on Fig. 2

Please see our response above.

On animal experiments please state clearly how many cohorts of animals were used

Thank you for pointing this out. The animal experiments were performed in two separate cohorts. We have now clarified this in the text (Lines 548-549).
